# The clinical efficacy of psychological interventions for bipolar depression: a systematic review and individual patient data (IPD) meta-analysis

**DOI:** 10.1017/S0033291725001023

**Published:** 2025-05-22

**Authors:** Sakir Yilmaz, Anna Huguet, Steve Kisely, Sanjay Rao, JianLi Wang, Molly Price, Richard Morriss, Maree Inder, Tania Perich, Kim Wright

**Affiliations:** 1 Department of Psychology, Abdullah Gul University; 2 Department of Psychology, University of Exeter; 3 Virgili Departament de Psicologia, Universitat Rovira I; 4 IWK Health Centre; 5 Department of Community Health and Epidemiology, and of Psychiatry, Dalhousie University; 6 Metro South Addiction and Mental Health Service; 7 Greater Brisbane Clinical School, Medical School, Princess Alexandra Hospital Southside Clinical Unit, The University of Queensland; 8 Faculty of Medicine, Psychiatry, University of Ottawa; 9 Canadian Association of Cognitive Behavioural Therapy; 10 University of Cardiff; 11 University of Plymouth; 12 School of Medicine, Unit of Mental Health and Neuroscience, University of Nottingham; 13 Biomedical Research Centre, Nottingham National Institute of Health Research; 14 NIHR MindTech Health Technology Collaborative; 15 NIHR Applied Research Collaboration East Midlands; 16 Department of Psychological Medicine, University of Otago; 17 School of Psychology, Western Sydney University; 18 Translational Health Research Institute, Western Sydney University

**Keywords:** Bipolar depression, psychological interventions, individual patient data meta-analysis

## Abstract

Unlike conventional meta-analyses, individual patient data (IPD) meta-analysis assesses moderator variables at the level of each participant, which generates more precise and biased estimates. The objective of this study was to investigate whether psychological therapy reduces depression symptoms in people with Bipolar I and II disorders and examine whether baseline depression has a moderating effect on treatment outcomes. Through the use of several electronic databases, a systematic search was conducted. Eligible studies were randomized controlled trials evaluating a psychological intervention for adults diagnosed with Bipolar I or II disorder. Titles and abstracts were screened, followed by full texts. The authors of the included studies were asked to provide IPD from their trials. A multilevel model approach was used to analyze the data. From the 7552 studies found by our searches, six studies with 668 study participants were eligible. Intervention significantly reduced depression scores. There was a significant association between baseline depression and post treatment depression scores. There was no statistically significant interaction between condition allocation and baseline depression score. When IPD from the two most comparable studies were analyzed, CBT had reduced depression scores relative to the comparator condition. The study included patient data from only six studies which were heterogeneous in terms of intervention type, outcome measure, and comparators. Overall, the psychological interventions tested significantly reduced bipolar depression scores. There was no evidence of moderation by baseline depression scores.

## Introduction

Bipolar disorder (BD) is categorized as a lifelong, clinically severe mood disorder that has episodic onsets. Late adolescence to early adulthood is the most common time of onset for BD (Joyce, 1984). A substantial number of adults with BD, however, start experiencing symptoms before they reach adulthood (Chengappa et al., 2003). Although the majority report both mania and depression, depressive symptoms are more frequent and last longer than periods of elevated mood or mixed symptoms (e.g., Miller, Dell’Osso, & Ketter, 2014). These major depressive episodes can last from weeks to months and even years, with potentially severe consequences for the person’s employment, financial situation, and quality of life (Abraham et al., 2014; Gilbert & Marwaha, 2013; McMorris, Downs, Panish, & Dirani, 2010; Martín-Subero et al., 2014, Frey, Kaya, Adeniyi, & McCabe, 2023). In the longer term, subsyndromal depression is the main burden of the disease (Judd et al., 2002; Judd et al., 2003). In addition to long periods of depressive symptoms, individuals with BD are at increased risk of some chronic physical health problems, early mortality (Chan et al., 2021), and death through suicide (Simon et al., 2007).

There are evidence-based pharmacological approaches for the treatment of recurrent bipolar depression (e.g., NICE, 2014; Yatham et al., 2018). However, not all individuals with bipolar depression wish to take medication or are able to tolerate it, and despite optimal pharmacotherapy, many people with BD continue to suffer from depressive symptoms.

Psychological interventions may offer an alternative approach to improve outcomes for people with BD as an adjunct to medication. These interventions focus on reducing symptoms while enhancing overall well-being and functional outcomes (Kaya & McCabe, 2022). While some reviews have concluded that psychological approaches are effective in reducing bipolar depression (Chiang et al., 2017; Yilmaz et al., 2022), others have not (Bond & Anderson, 2015; Chatterton et al., 2017). There are a number of possible explanations for this discrepancy, including differences in the sets of studies analyzed. We argue that two particular characteristics of study design are of importance in evaluating the efficacy of psychological treatments for bipolar depression but may be overlooked within meta-analyses. First, if the therapy protocol does not primarily target reduction in depression, we might expect that any effect on depression may be diluted. For example, a therapy protocol that teaches skills to manage co-occurring substance use may be less effective in reducing depression than one that purports to directly modify psychological factors known to contribute to depression risk and maintenance. Second, if the study does not select participants who are currently depressed, the potential to show improvement in depressive symptoms is constrained. In a previous meta-analysis (Yilmaz et al., 2022), we found that relatively few studies targeted acute depression specifically, and only one required the participant to be acutely depressed, thus limiting the potential of our analysis to draw conclusions about the impact of therapy on acute bipolar depression. If depression level at intake does affect the extent to which therapy can show benefit, one might expect intake depression level to moderate treatment effect, such that therapy is more beneficial for those who are more depressed. This moderation effect was not found in our aggregate meta-regression analysis; however, meta-regression is limited in that whole-study effect sizes are the unit of analysis, rather than data from individual participants. To address this, we conducted an individual patient data meta-analysis (IPD-MA), allowing us to both replicate the evaluation of the efficacy of psychological therapy in treating depressive symptoms and conduct an analysis of the moderating effect of depression symptoms upon treatment response, both using disaggregated data.

While conventional meta-analysis examines moderator variables at the level of the individual study, IPD-MA does so at the level of the individual participants, resulting in more precise and less biased estimates of the effect than conventional meta-analysis and offering the potential for more complex models of covariates to be constructed (Burke, Ensor, & Riley, 2016; Debray et al., 2013; Riley et al., 2020). In meta-analysis techniques, heterogeneity plays a crucial role and has a significant impact on findings. True variations in participants, interventions, cointerventions, outcomes, assessments, contexts, and a plethora of other variables that change throughout the sets of data, studies, and participants might represent the cause of heterogeneity (Ioannidis, 2008). One specific issue that arises from heterogeneous data in meta-analyses is the challenge of synthesizing results from studies with different methodologies or populations. Heterogeneity in data can lead to difficulties in interpreting the overall effect size and can affect the validity of the meta-analysis results (Borenstein, Hedges, Higgins, & Rothstein, 2011). Another issue is the potential for statistical heterogeneity, which occurs when there is variability in effect sizes beyond what would be expected by chance alone. This can stem from differences in study design, measurement tools, or participant characteristics (Higgins & Thompson, 2002). Furthermore, heterogeneity can impact the choice of statistical models used in the meta-analysis. If the data are highly heterogeneous, traditional fixed-effects models may not be appropriate, and researchers may need to resort to random-effects models or other methods to account for the variability (Borenstein, Hedges, Higgins, & Rothstein, 2011). Lastly, heterogeneity can affect the generalizability of meta-analytic findings. If the included studies vary widely in terms of their populations or methodologies, it may be challenging to draw conclusions that are applicable across different contexts or settings (Ioannidis, 2008).

In addressing these issues, researchers must carefully assess and account for heterogeneity through sensitivity analyses, subgroup analyses, or by considering alternative statistical approaches to ensure the robustness and reliability of their meta-analytic findings. Individual patient-level data meta-analysis is one means of addressing clinical and methodological diversity.

Here, we report the outcome of our IPD-MA of studies of psychological therapies where bipolar depression level was measured at intake and post-treatment. We hypothesized that psychological therapies are effective in reducing depressive symptoms for people living with Bipolar I and II disorders. We also hypothesized that those with higher levels of depression at baseline stand to benefit the most from receiving the treatment.

## Methods

### Protocol and registration

Our study protocol was registered on the PROSPERO database (CRD42019148696). Our report is written in accordance with the Preferred Reporting Items for Systematic Reviews and Meta-Analyses of Individual Participant Data (PRISMA) guideline (Stewart et al., 2015).

Approval for this secondary data analysis was obtained from the University of Exeter Department of Psychology Research Ethics Committee (eCLESPsy002050). Researchers contributing data were asked to ensure that doing so was within the scope of their existing ethical approvals.

The selection criteria and selection process for studies have been previously reported (Yilmaz et al., 2022), but they are summarized below. Besides the searches described in Yilmaz et al’s study, an updated database search was conducted in Yilmaz et al., 2022 to make sure that we were not missing any studies published after the final search of our earlier study was conducted and had the opportunity to be contacted by us to send the IPD from their study.

### Eligibility criteria

Studies had to evaluate the efficacy or effectiveness of psychological interventions for adults with Bipolar I or II disorders in a randomized controlled trial using a validated diagnostic instrument (i.e., SCID, SADS, CIDI, PSE-10 SCAN, and MINI) or clinical diagnosis by a qualified individual. Relevant psychological interventions were defined as follows: “interpersonal or informational activities, techniques, or strategies that target biological, behavioral, cognitive, emotional, interpersonal, social, or environmental factors with the aim of improving health functioning and well-being” (Institute of Medicine, 2015). There was no restriction on whether the psychological intervention was the primary or adjunctive therapy (i.e., delivered in conjunction with ongoing medication as part of usual care). The following control conditions were included: treatment/care as usual, wait-list, active control, and placebo. Studies were required to assess depression symptom levels at pre- and post-treatment, using continuous or categorical scales, based on researchers’ or clinician’s ratings. Participants in the studies were required to be aged 16 and older. English-language publications and studies published between 1952 and 2020 were eligible.

### Study identification and selection process

As part of the search strategy, terms were included for BDs (e.g., manic depression; mania), depression (e.g., depressive), therapy (e.g., psychotherapy; behav* activation), and randomized control trials (e.g., random allocation; randomization). Each database has its own set of subject headings; our search terms were determined based on these. A search was conducted using the following databases. Cochrane Controlled Register of Trials (1996), MEDLINE (1966 onwards) (see online Supplementary Material- Figure S1 Search strategy for MEDLINE), EMBASE (1980 onwards), PsycINFO (1974 onwards), Scopus, Web of Science and Clinical Trials Registries (listed at: https://www.hhs.gov/ohrp/international/clinical-trial-registries/index.html). The reference lists of relevant systematic reviews and meta-analyses were also reviewed for potential eligibility.

EndNote software was used to compile all studies retrieved. A randomly selected set of 20% of titles/abstracts retrieved by the search strategy was independently screened by two reviewers (SY, KB) who then calculated the degree of agreement between the reviewers (Kappa = 0.82). Disagreements between the two reviewers were discussed and resolved with the assistance of a third reviewer (AH) as required. Upon reaching an agreement, one reviewer (SY) screened the remaining studies. The full text of potentially eligible studies was retrieved after the full list of titles and abstracts had been screened. In cases where we were unable to locate the full-text article, study authors were contacted to request the article. Following the same procedure as previously, two reviewers (SY, KB) screened full articles. When discrepancies were identified, a third reviewer (AH) was involved.

### Data collection and data items

As reported in our pairwise meta-analysis (Yilmaz et al., 2022), one randomly selected study was used as a pilot test by two reviewers (SY and AH) to develop a data extraction sheet. Using the extraction form, data were extracted from included studies. The information for all studies in the review was extracted independently by two reviewers (SY and KB). In the event of discrepancies between the reviewers, they were discussed and resolved between them. In the event that a consensus could not be reached, a third reviewer (AH) was involved.

Individual data about the following variables were requested from the authors of the included studies: scores on depression outcomes at baseline and follow-up points; clinical information about patients at baseline (such as BD subtype, number of previous episodes, comorbidities); demographic information (age, sex, ethnicity, education level, socioeconomic status); therapy attendance data (number of sessions attended, drop out status [did/did not drop out of therapy]); and a copy of the therapy protocol if available.

The authors were contacted by email after a few weeks if they did not respond to the initial request, and attempts were also made to contact coauthors. In the absence of a response, we regarded the data as unavailable and excluded the study from the analysis. A data sharing agreement (see online Appendix 1 for details) was entered into with authors agreeing to provide the data. Individual databases were aggregated into one large IPD database which was then checked for accuracy.

The posttreatment outcome was defined as the period immediately after the end of the acute treatment phase but not later than 3 months after the end of the acute treatment phase.

### Quality of evidence

A Cochrane Collaboration risk of bias tool (Higgins et al., 2011) was used by two reviewers (SY, MP) to assess the quality of selected studies. The discrepancies were reviewed by a third reviewer (AH). The following items were assessed: selection bias; performance bias; detection bias; attrition bias; reporting bias; and other bias (intention to treat analysis and group similarity at baseline, checks of the training of the therapist, manualization of the therapy and whether fidelity to the therapy method had been assessed through rating tapes of all or only a subset of sessions). Each study was subjected to a risk assessment regarding the bias arising from the individual items. Risk of bias can be categorized as low, unclear, or high.

As part of the Grading of Recommendations Assessment, Development and Evaluation (GRADE) framework (Schünemann, Brożek, Guyatt, & Oxman, 2013), a pooled effect estimate was estimated for each outcome. With GRADE, quality levels are categorized as very low, low, moderate, and high based on a variety of domains. The quality rating of a study is affected by the presence of study limitations, inconsistency, indirectness, imprecision, and publication or reporting bias, as outlined in this framework. Based on GRADE’s framework, one reviewer (KW) with subject matter expertise evaluated the overall quality of the evidence, and a second reviewer (SY) verified the decision (see Table 2).

### *IPD analysis approach* ^*1*^

In total, the data were received from six studies. Of these, two studies used the Beck Depression Inventory (BDI), two the Montgomery-Asberg Depression Rating Scale (MADRS), one the Beck Depression Inventory II (BDI-II), and the Hamilton Depression Rating Scale (HAM-D) as the main depression outcome measure. The scores of all measures pre and posttreatment were converted to HAMD scores using published algorithms in order to ensure maximum comparability (Furukawa et al., 2019; Leucht et al., 2018; see table 1). Table 3 summarizes the characteristics of the studies included in our main analysis.

**Table 1.** Converted HAM-D baseline mean and SD scores across studies

Study ID	Mean	SD
Van Dijk, Jeffrey, and Katz (2013)	10.73	8.67
Perich et al. (2013)	7.05	6.42
Isasi, Echeburúa, Limiñana, and González-Pinto (2010)	9.39	6.28
Morriss et al. (2016)	7.67	7.27
Lam et al. (2003)	8.68	8.33
Inder et al. (2015)	8.12	7.15

**Table 2.** GRADE evidence profile

		Quality assessment					Summary of finding		
Comparison	Number of studies	Risk of bias	Inconsistency	Indirectness	Imprecision	Publication bias	Number of individuals	Comparator	Quality
Psychological treatment versus control group at posttreatment	4	Serious limitations	Substantial heterogeneity	Very serious	No serious	Undetected	293	290	Low
CBT versus control group at posttreatment	2	Serious limitations	No evidence of significant heterogeneity	Very serious	No serious	Undetected	66	64	Low

Abbreviation: CBT, cognitive behavior therapy.

**Table 3.** Summary of the characteristics of the included studies

Studies, country	Primary diagnosis	Study setting	Primary intervention target	Depression status at baseline	Number of individuals randomized	Length of follow-up (in months	Duration of acute treatment (in weeks)	Duration of treatment sessions (in minutes)	Number of treatment sessions	Treatment outcome measure	Treatment group	Treatment modality	Comparator group
Inder et al. (2015) New Zelland	BSNS	Unclear/Unspecified	Other	Not specified	100	18	Not stated	Not stated	Not stated	MADRS	IPSRT	Individual therapy	Active control
Van Dijk, Jeffrey, and Katz (2013), Canada	BSNS	Outpatient	Unclear/unspecified	Not specified	26	N/A	12	90	12	BDI-II	DBT	Group therapy	Waiting list
Perich et al. (2013), Australia	BSNS	Outpatient	Relapse prevention	Not depressed	95	12	8	120–150	8	MADRS	MBCT	Group therapy	Usual care
Morriss et al. (2016), Uk	BSNS	Unclear/unspecified	Unclear/unspecified	Not depressed	304	24	26	120	21	Ham-D	Psychoeducation	Group therapy	Placebo
Lam et al. (2003), Uk	Bipolar I disorder	Outpatient	Relapse prevention	Not depressed	103	6	24	60	12–18	BDI	CBT (Adapted version of Cognitive therapy)	Individual face to face therapy	Usual care
Isasi, Echeburúa, Limiñana, and González-Pinto (2010), Spain	Bipolar I disorder	Outpatient	Unclear/unspecified	Not depressed	40	12	20	90	20	BDI	CBT (Adapted version of Cognitive therapy)	Individual face to face therapy	Usual care

Abbreviations: BSNS, Bipolar subtype(s) not specified; MADRS, Montgomery-Asberg Depression Rating Scale; BDI, Beck Depression Inventory; BDI-II, Beck Depression Inventory II; HAM-D, Hamilton Depression Rating Scale; MBCT, mindfulness-based cognitive therapy; CBT, cognitive behavior therapy; DBT, dialectical behavior therapy; IPSRT, interpersonal and social rhythm therapy.

Individual databases were aggregated into one large IPD database which was then checked for accuracy against the published manuscripts. Following this, to permit an intention-to-treat analysis, IBM SPSS version 28.0.1.0 was used to impute the missing outcome data of depression scores using multiple imputation algorithm on the assumption of missing-at-random (MAR). In this method, a number of datasets are generated and analyzed separately using the selected model, and their results are then combined. As opposed to using incomplete samples or mean imputation, multiple imputation is more likely to produce results that are less biased (Donders, van der Heijden, Stijnen, & Moons, 2006). Overall, 0.59% of pretreatment and 25.29% of posttreatment data were missing. A 100-times imputing procedure was performed using complete patient and study characteristics, namely baseline depression score, age, and gender, as predictor variables. A sensitivity analysis was also conducted based on only those cases with data being available (complete case analysis) which is based on the missing not at random (MNAR) assumption.

By conducting a one-stage IPD-MA, all patient data from all studies were combined, with individuals nesting within studies. Multilevel linear mixed models were used and clustered at the study level to be able to detect and control for the unobserved heterogeneity between studies. Baseline depression score, age, and gender were included as covariates.

As part of the primary analysis, we examined how condition allocation affected depression outcomes. Afterward, we examined whether baseline depression moderated treatment outcomes. The outcome variable was the HAM-D posttreatment score, while condition allocation, baseline HAM-D depression score, age, and gender were included as predictors. An interaction between baseline depression scores and treatment outcomes was incorporated into the multilevel linear regression model to determine if baseline depression scores were a moderator of treatment outcomes.

Data were analyzed using IBM SPSS version 28.0.1.0. A multilevel modelling approach was used to address both hypothesis 1, which predicted that psychological therapies are effective in reducing depressive symptoms for people living with Bipolar I and II disorders, and hypothesis 2, which predicted that treatment will be most effective for individuals with higher levels of depression. Multilevel modelling was used because individuals (level 1) were nested within studies (level 2). HPS scores were included as a level 2 predictor. In terms of data preparation, it is essential that fixed effects in multilevel models be easily interpreted within the context of the research goals.

**Figure 1.**
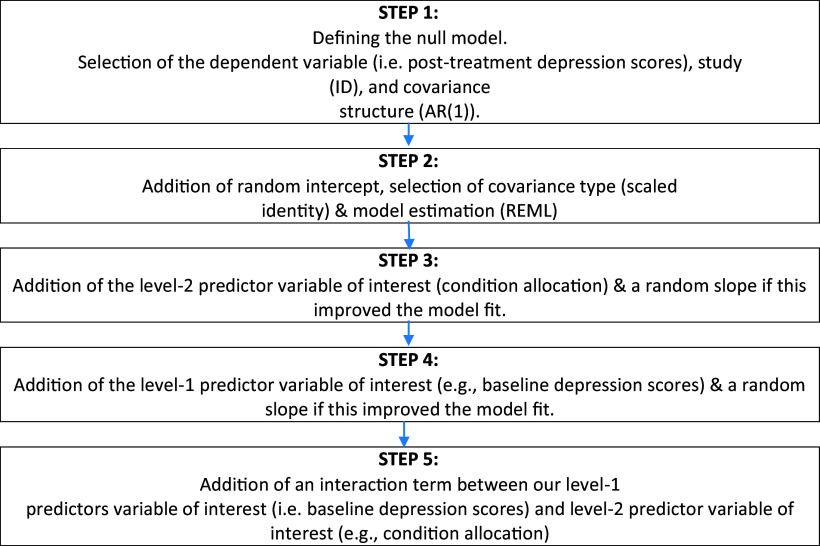
A schematic representation of the five steps involved in constructing a multilevel linear model. *Notes.* ID, identification; REML, restricted maximum likelihood.

Our approach to multilevel modelling was informed by guidance from Heck, Thomas, and Tabata (2014). Using The akaike information criterion (AIC) and restricted log-likelihood estimations, we compared successive models and retained elements according to whether they improved model fit. Defining a null model was the first step. The study (ID) and the type of covariance structure (AR(1)) were entered. AR (1) refers to a first-order autoregressive structure with homogeneous variances. A random intercept was then allowed to vary between participants in step 2. Scaled identity was selected as the covariance type as a constant variance was present in this structure and it was assumed that there was no correlation between any of the elements. As the model estimation parameter, restricted maximum likelihood was selected because our sample was small in terms of number of level 2 units. In step 3, the level 2 predictor variable of interest (condition allocation) was introduced. A random slope was also added to the model in this step. In step 4, the level 1 predictor variable of interest (e.g., base depression scores) was introduced. A random slope was also added to the model in this step. In step 5, we included an interaction term between level 1 predictor variable of interest (e.g., baseline depression scores) and our level 2 predictor variable of interest (condition allocation). An overview of each model’s development from the null model is shown in Figure 1.

## Results

### Study selection

**Figure 2.**
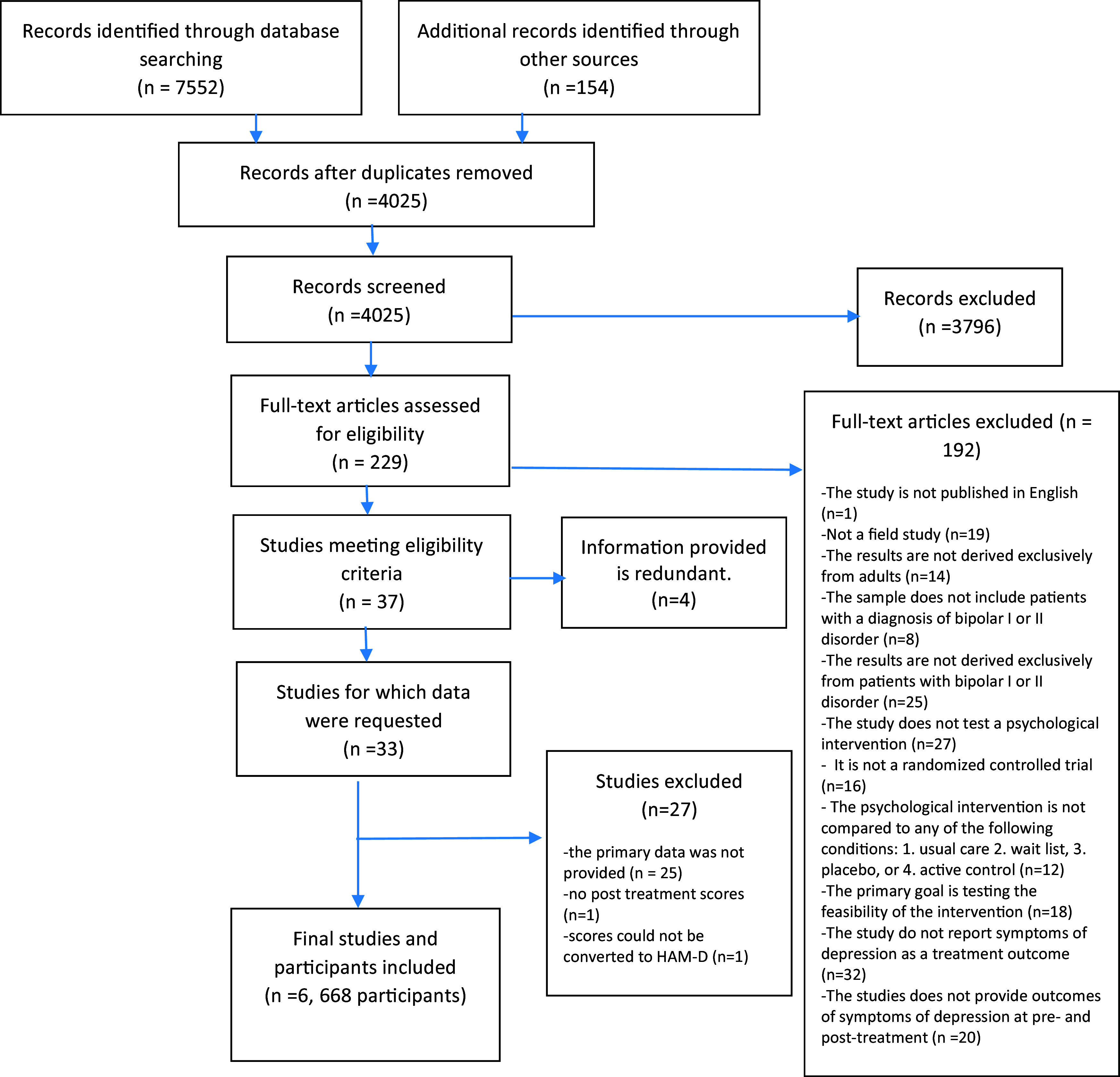
Flow diagram from record identification to study inclusion.

A total of 7552 studies were identified through database searches and search results of relevant systematic reviews and meta-analyses. A total of 3681 duplicates were removed. Following the screening of titles and abstracts, a further 3789 studies were excluded. A full-text review of 229 studies was performed, and of these, 192 studies were excluded due to their failure to meet the inclusion criteria. Four of the remaining 37 studies were also excluded as they provided redundant information; therefore, data were requested from the authors of 33 studies. Following this, 27 studies were excluded due to various reasons, including: (1) the primary IPD were not provided (see online Supplementary Material – Tables); (2) posttreatment scores were not available; and (3) scores were not be able to be converted to HAM-D scores. The remaining six studies were included in the analysis (see Figure 2).^2^ ^,^^3^

### Study and patient characteristics

Five types of psychological therapies were identified: cognitive behavioral therapy (CBT), psychoeducation, interpersonal and social rhythm therapy (IPSRT), mindfulness-based cognitive therapy (MBCT), and dialectical behavior therapy (DBT). Studies were conducted in five countries: Australia, New Zealand, Canada, Spain, and UK. Recruitment was from out-patient settings for four studies; for two studies, setting was unclear. Two studies examined individual face to face CBT; one psychoeducation (group therapy); one DBT (group therapy); one MBCT (group therapy); and one IPSRT (individual therapy). Control groups consisted of treatment as usual (3), waiting list (1), placebo (1), and active control (1). The number of participants in the studies ranged from 26 to 304. Overall follow-up duration ranged from 12 to 24 months. The duration of acute treatment ranged from 8 to 26 weeks. Acute depression was not the primary target for any of the interventions (see Table 3 for further details). Two of the studies identified relapse prevention as the primary intervention target (we conceptualized “primary intervention target” as the stated focus of the treatment, rather than the stated primary outcome), while in three it was unclear/unspecified, and one study identified other.

A total of 668 participants across the six studies were included in the IPD-MA, 334 from the intervention condition and the remainder from the control condition. Of the 668 study participants, the mean (SD) age was 40.8 (12.49) years, while 254 (38%) of 666 were male and 414 (62%) were female. The mean (SD) baseline scores were 6.38 (5.10) on HAM-D, 18.60 (5.48) on BDI-II, 13.81 (10.03) on MADRS, and 12.84 (9.66) on BDI.

### Study quality (Risk of bias)

**Figure 3.**
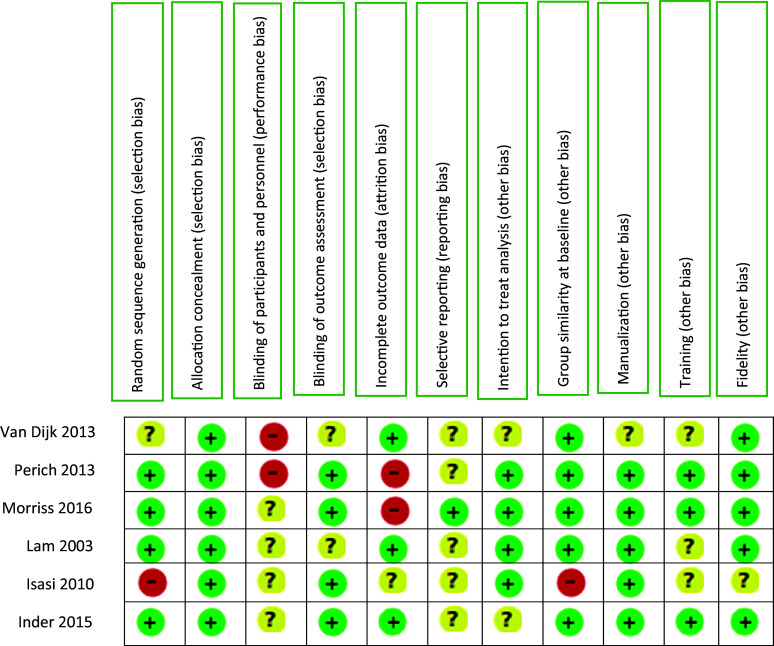
Cochrane risk of bias assessment for included RCTs.

Figure 3 shows risk of bias of included studies. Four studies were at low risk of bias for random sequence generation, whereas one study was at high risk and one did not report sufficient information to allow assessment. There was a low risk of bias for allocation concealment in all studies. Two studies were not able to blind participants and personnel, while the rest were unclear. The blinding of outcome assessments was possible in four studies, while it was unclear in the remaining studies. There were four studies that used intention-to-treat analysis, but the remainder were unclear. For group similarity at baseline, one study was at high risk of bias. The risk of bias for selective reporting was low in only one study, while the risk was unclear in the others. For five studies, manualization was at a low risk of bias, although one study was unclear. Two studies were at a low risk of bias for training, although the rest were unclear. Four studies were at a low risk of bias for fidelity, although the rest were unclear.

Overall, the quality of evidence used to draw conclusions in this study was low (see Table 2)

### One-stage IPD meta-analysis findings

Two sets of analyses were conducted. In the first analysis, we included all six studies. Acknowledging the heterogeneity in study characteristics in this group of studies, in the second set of analyses, we included the only subset of studies that were highly comparable in terms of intervention and comparator condition, namely the two studies evaluating CBT versus usual care. A full breakdown of these results can be found in the online Supplementary Material – Tables.

#### Analysis of the complete study set

We determined that the best-fitting model included a random intercept. When we added condition allocation as a factor in the model, results showed a significant effect of intervention on depression scores γ = 1.86 CI [.58, 3.15], SE = .65, p < .005. When adding a random slope for condition allocation, it improved the fit of the model, so this was included in the final model.

Baseline depression score was then added to the model. The results showed that the baseline depression score was significantly associated with the posttreatment depression score such that a higher depression score at baseline was associated with a higher depression score at posttreatment γ = .43 CI [.34, .51], SE = .04, p < .000. The effect of the intervention remained significant. When adding a random slope for baseline depression, it improved the fit of the model, so this was included in the final model.

The interaction between condition allocation and baseline depression was added to the model. The result showed that the interaction was not statistically significant γ = .05 CI [−.28, .38], SE = .17, p = .762. Following that, age and gender were added to the model, and this did not change the pattern of findings.

The pattern of results from the complete-case sensitivity analysis remained as for the intention-to-treat analysis.

#### Analysis of a subset of comparable studies

The above analyses were conducted a second time, in this case including only two studies (Isasi, Echeburúa, Limiñana, & González-Pinto, 2010; Lam et al., 2003) as these were the only subsets comparable in terms of their intervention and comparator conditions. We determined that the best-fitting model included a random intercept.

A significant effect of CBT on depression scores relative to the comparator condition (treatment as usual) was observed when condition allocation was included in the model γ = 2.72 CI [.05, 5.39], SE = 1.36, p = .046.

The baseline depression score was then included in the model. The main effect of baseline depression score was significant, such that higher depression score at baseline was associated with a higher depression score at posttreatment γ = .61 CI [.45, .78], SE = .08, p < .001.

An interaction between condition allocation and baseline depression was included in the model. The interaction between condition allocation and baseline depression did not predict outcome γ = .03 CI [−.14, .20], SE = .09, p = .752. This was followed by the addition of age and gender to the model, and this did not change the pattern of findings.

The pattern of results from the complete case sensitivity analysis remained as for the intention-to-treat analysis. We were not able to conduct an analysis of follow-up data as insufficient follow-up data were available.

## Discussion

This study used IPD-MA to explore whether psychological therapies can reduce the symptoms of depression among people living with Bipolar I and II disorders and whether baseline depression moderates treatment outcomes. By examining patient-level characteristics that may act as moderators of treatment effects rather than only study-level effects, we were able to move beyond the meta-analysis of aggregate data that we conducted previously (Yilmaz et al., 2022).

We found a significant effect of intervention on posttreatment depression scores in this IPD-MA. The findings are in line with the results of our and other authors’ previous meta-analyses of aggregate data (Miklowitz et al., 2021; Oud et al., 2016; Yilmaz et al., 2022). These positive findings also applied to our subset analysis of IPD. Some meta-analyses have not found a benefit of psychological therapies for bipolar depressive symptoms (Bond & Anderson, 2015; Chatterton et al., 2017; Ye et al., 2016). Differences across meta-analyses in choice of primary end point, type of therapies included, and whether therapy was required to be adjunctive to medication may explain this discrepancy.

In our analysis, we examined the impact on depression posttreatment, within a tightly defined timeframe. We also did not limit study samples to those already taking mood-stabilizing medication. Our findings support the use of manualized psychological therapy for the reduction of depression symptoms. Consistent with our previous meta-analysis of aggregate data utilizing a larger set of studies (Yilmaz et al., 2022), we were able to draw this conclusion most confidently with respect to CBT: in the current analysis, this was the only therapy type where data were available from more than one study, permitting a subgroup analysis. In our aggregate meta-analysis, effect sizes across trials for CBT were particularly consistent. In terms of implications for clinical practice, our finding is not that CBT is more effective than other therapies in reducing bipolar depression; instead, within the group of trials we studied, the number and homogeneity of CBT trials affords relative confidence in this as an evidence-based intervention for reducing bipolar depressive symptoms by the end of treatment.

In this study, we examined baseline depression as a potential moderator of intervention effect. Overall, we found that those who began therapy with more severe depression symptoms tended to have higher depression scores at the end of treatment, and the interaction between condition allocation and baseline depression did not predict outcome in the full study set, nor in the subset of two CBT studies. This is contrary to our prediction that those with higher levels of depression stand to benefit the most from receiving the treatment. Indeed, the literature on unipolar depression suggests those with greater depression severity show a greater response to psychological therapy when therapy is compared to passive control conditions (Driessen, Cuijpers, Hollon, & Dekker, 2010; van Bronswijk et al., 2019). Our finding may reflect the composition of the sample and the focus of the therapy protocols: none of the included studies explicitly stated that reduction of acute depression was their primary target; therefore, the therapy protocols may not have focused primarily upon targeting depression, weakening the potential to show an effect on depression symptoms. Relatedly, because participants were not required to be in a major depressive episode at baseline in the trials we included, our findings do not speak to the efficacy of psychological therapy for acute bipolar depression, nor what moderates any effect. Meta-analysis of trials of psychological therapies that are explicitly designed to target acute depression would be needed for this purpose. While a small number of such trials have been conducted, data from these were not available for the current analysis.

### Limitations

As is often the case for IPD-MAs, our findings have been influenced by availability bias: 25 potentially relevant studies were excluded because the authors were unable to provide primary data.

Furthermore, there was considerable variation in intervention type, outcome measure, and comparator among the studies included in the analysis, which did not allow us to make all of the desired comparisons and introduced a number of potential sources of heterogeneity. Given the inconsistent reporting of factors such as previous mania or other relapse history, bipolar I or bipolar II disorder, and medication among the studies reported in the meta-analysis, the sources of heterogeneity remain unclear. The overall outcomes in our study may mask differences between therapies in terms of their effectiveness in treating bipolar depression, and the extent to which this depends upon baseline depression level. However, our findings were similar in the subset analysis that was restricted to studies of CBT. Heterogeneity was reduced, suggesting that the type of intervention was one source of heterogeneity among the studies included in the IPD-MA.

A consistent measure of bipolar depression was lacking among studies, which made it challenging to synthesize the data. In our study, we converted scores to HAM-D scores, which may introduce a potential source of error. Future studies should attempt to harmonize outcome measurement to reduce error in IPD-MAs that include them.

All studies took place in countries with high incomes. As a consequence, it may not be possible to generalize these results to other countries with lower or middle incomes.

We used the MAR assumption when we applied multiple imputation. We could only impute missing values based on the baseline variables present, which were limited. We conducted a complete case analysis to check whether the pattern of findings appeared robust, and this was the case, however, this cannot inform us as to whether the application of the MAR assumption was correct. A separate but related issue is that we used the depression score at baseline to impute the depression score at posttreatment, which may confound the exploration of their association. However, because this association remained significant in the complete case analyses, confidence in this aspect of the study is increased.

### Implications and conclusion

Although systematic investigations of psychological treatments for BD have emerged over the last few decades, there is no strong consensus regarding which forms of psychotherapy lead to the greatest clinical improvement in BD and for which phases of the condition. The findings of our study are in accordance with previous research, which supports the use of psychological interventions to reduce depression symptoms in BD as part of relapse prevention or maintenance therapy, especially CBT. Our findings do not indicate that psychological therapy efficacy varies according to baseline depression severity; however, this conclusion cannot be considered definitive because of substantial variation between trials in therapy type, comparator, and treatment target, as well as other potential confounders indicated by high heterogeneity in the IPD-MA. In order to address this issue, trials of psychological therapy for BD should state the therapeutic target(s) of their protocol as well as clearly defining the target population, for example in terms of depression status. This will allow more accurate estimates of the treatment effects that are possible for those experiencing acute bipolar depression.

## Supporting information

Yilmaz et al. supplementary material 1 Yilmaz et al. supplementary material

Yilmaz et al. supplementary material 2 Yilmaz et al. supplementary material

## Footnotes

1 We did not analyze aggregate data given our recently published pairwise meta-analysis (Yilmaz et al., 2022).

2 Because of the additional search mentioned in Section title “Protocol and registration”, the numbers in the flow diagram differ from those in the previous study.

3 We identified four studies with a lower age limit of 15, which was below our criterion of 16. One of these (Inder et al., 2015) was eligible according to all other criteria. As this study included only one participant aged below 16 (1% of the sample), the decision was made to include this study in the analyses in order to maximize inclusion of relevant data.

## Supplementary material

The supplementary material for this article can be found at http://doi.org/10.1017/S0033291725001023.

## References

[r1] Abraham, K. M., Miller, C. J., Birgenheir, D. G., Lai, Z., & Kilbourne, A. M. (2014). Self-efficacy and quality of life among people with bipolar disorder. The Journal of Nervous and Mental Disease, 202(8), 583–588. 10.1097/NMD.0000000000000165 25010107 PMC4133989

[r3] Bond, K., & Anderson, I. M. (2015). Psychoeducation for relapse prevention in bipolar disorder: A systematic review of efficacy in randomized controlled trials. Bipolar Disorders, 17(4), 349–362. 10.1111/bdi.12287 25594775

[r4] Borenstein, M., Hedges, L. V., Higgins, J. P.*, &* Rothstein, H. R*. (*2011*).* Introduction to meta-analysis. John Wiley & Sons

[r6] Burke, D. L., Ensor, J., & Riley, R. D. (2016). Meta-analysis using individual participant data: One-stage and two-stage approaches, and why they may differ. Statistics in Medicine, 36(5), 855–875. 10.1002/sim.7141 27747915 PMC5297998

[r7] Chan, J. K. N., Wong, C. S. M., Yung, N. C. L., Chen, E. Y. H., & Chang, W. C. (2021). Excess mortality and life-years lost in people with bipolar disorder: An 11-year population-based Cohort Study. Epidemiology and Psychiatric Sciences, 30, e39. 10.1017/s2045796021000305 34044906 PMC8193965

[r8] Chatterton, M. L., Stockings, E., Berk, M., Barendregt, J. J., Carter, R., & Mihalopoulos, C. (2017). Psychosocial therapies for the adjunctive treatment of bipolar disorder in adults: Network meta-analysis. The British Journal of Psychiatry, 210(5), 333–341. 10.1192/bjp.bp.116.195321 28209591

[r9] Chengappa, K. N., Kupfer, D. J., Frank, E., Houck, P. R., Grochocinski, V. J., Cluss, P. A., & Stapf, D. A. (2003). Relationship of birth cohort and early age at onset of illness in a bipolar disorder case registry. The American Journal of Psychiatry, 160(9), 1636–1642. 10.1176/appi.ajp.160.9.1636 12944339

[r10] Chiang, K. J., Tsai, J. C., Liu, D., Lin, C. H., Chiu, H. L., & Chou, K. R. (2017). Efficacy of cognitive-behavioral therapy in patients with bipolar disorder: A meta-analysis of randomized controlled trials. Plos One, 12(5). 10.1371/journal.pone.0176849 PMC5417606 28472082

[r11] Debray, T. P., Moons, K. G., Abo-Zaid, G. M., Koffijberg, H., & Riley, R. D. (2013). Individual participant data meta-analysis for a binary outcome: One-stage or two-stage? Plos One, 8(4). 10.1371/journal.pone.0060650 PMC3621872 23585842

[r12] Donders, A. R., van der Heijden, G. J., Stijnen, T., & Moons, K. G. (2006). Review: A gentle introduction to imputation of missing values. Journal of Clinical Epidemiology, 59(10), 1087–1091. 10.1016/j.jclinepi.2006.01.014 16980149

[r13] Driessen, E., Cuijpers, P., Hollon, S. D., & Dekker, J. J. M. (2010). Does pretreatment severity moderate the efficacy of psychological treatment of adult outpatient depression? A meta-analysis. Journal of Consulting and Clinical Psychology, 78(5), 668–680. 10.1037/a0020570 20873902

[r14] Frey, A.-L., Kaya, M. S., Adeniyi, I., & McCabe, C. (2023). Anhedonia in relation to reward and effort learning in young people with depression symptoms. Brain Sciences, 13(2), 341. 10.3390/brainsci13020341 36831884 PMC9953984

[r15] Furukawa, T. A., Reijnders, M., Kishimoto, S., Sakata, M., DeRubeis, R. J., Dimidjian, S., … Cuijpers, P. (2019). Translating the BDI and BDI-II into the Hamd and vice versa with equipercentile linking. Epidemiology and Psychiatric Sciences, 29. 10.1017/s2045796019000088 PMC8061209 30867082

[r16] Gilbert, E., & Marwaha, S. (2013). Predictors of employment in bipolar disorder: A systematic review. Journal of Affective Disorders, 145(2), 156–164. 10.1016/j.jad.2012.07.009 22877965

[r17] Heck, R. H., Thomas, S. L., & Tabata, L.N. (2014). *Multilevel and longitudinal modeling with IBM SPSS* (2nd ed.). Routledge. 10.4324/9780203701249

[r18] Higgins, J. P., Altman, D. G., Gotzsche, P. C., Juni, P., Moher, D., Oxman, A. D., … Sterne, J. A. (2011). The cochrane collaboration’s tool for assessing risk of bias in randomised trials. BMJ, 343, d5928. 10.1136/bmj.d5928 22008217 PMC3196245

[r19] Higgins, J. P. T., & Thompson, S. G. (2002). Quantifying heterogeneity in a meta-analysis. Statistics in Medicine, 21(11), 1539–1558. 10.1002/sim.1186 12111919

[r20] Inder, M. L., Crowe, M. T., Luty, S. E., Carter, J. D., Moor, S., Frampton, C. M., & Joyce, P. R. (2015). Randomized, controlled trial of interpersonal and social rhythm therapy for young people with bipolar disorder. Bipolar Disorders, 17(2), 128–138. 10.1111/bdi.12273 25346391

[r21] Institute of Medicine. (2015). Psychosocial interventions for mental and substance use disorders: A framework for establishing evidence-based standards. The National Academies Press. 26203478

[r22] Ioannidis, J. P. A. (2008). Interpretation of tests of heterogeneity and bias in meta-analysis. Journal of Evaluation in Clinical Practice, 14(5), 951–957. 10.1111/j.1365-2753.2008.00986.x 19018930

[r23] Isasi, A., Echeburúa, E., Limiñana, J., & González-Pinto, A. (2010). How effective is a psychological intervention program for patients with refractory bipolar disorder? A randomized controlled trial. Journal of Affective Disorders, 126(1–2), 80–87. 10.1016/j.jad.2010.03.026 20444503

[r24] Joyce, P. R. (1984). Age of onset in bipolar affective disorder and misdiagnosis as schizophrenia. Psychological Medicine, 14(1), 145–149. 10.1017/s0033291700003147 6709780

[r25] Judd, L. L., Akiskal, H. S., Schettler, P. J., Coryell, W., Endicott, J., Maser, J. D., … Keller, M. B. (2003). A prospective investigation of the natural history of the long-term weekly symptomatic status of bipolar II disorder. Archives of General Psychiatry, 60(3), 261–269. 10.1001/archpsyc.60.3.261 12622659

[r26] Judd, L. L., Akiskal, H. S., Schettler, P. J., Endicott, J., Maser, J., Solomon, D. A., … Keller, M. B. (2002). The long-term natural history of the weekly symptomatic status of bipolar I disorder. Archives of General Psychiatry, 59 *(*6), 530–537. 10.1001/archpsyc.59.6.530 12044195

[r27] Kaya, M. S., & McCabe, C. (2022). Effects of COVID-19 on adolescent mental health and internet use by ethnicity and gender: A mixed-method study. International Journal of Environmental Research and Public Health, 19(15), 8927. 10.3390/ijerph19158927 35897302 PMC9331135

[r28] Lam, D., Watkins, E., Hayward, P., Bright, J., Wright, K., & Kerr, N., … Sham, P. (2003). A randomized controlled study of cognitive therapy for relapse prevention for bipolar affective disorder. Archives of General Psychiatry, 60(2), 145. 10.1001/archpsyc.60.2.145 12578431

[r29] Leucht, S., Fennema, H., Engel, R. R., Kaspers-Janssen, M., & Szegedi, A. (2018). Translating the HAM-D into the Madrs and vice versa with equipercentile linking. Journal of Affective Disorders, 226, 326–331. 10.1016/j.jad.2017.09.042 29031182

[r30] Martín-Subero, M., Berk, L., Dodd, S., Kamalesh, V., Maes, M., Kulkarni, J., … Berk, M. (2014). Quality of life in bipolar and schizoaffective disorder–a naturalistic approach. Comprehensive Psychiatry, 55(7), 1540–1545. 10.1016/j.comppsych.2014.05.009 24962450

[r31] McMorris, B. J., Downs, K. E., Panish, J. M., & Dirani, R. (2010). Workplace productivity, employment issues, and resource utilization in patients with bipolar I disorder. Journal of Medical Economics, 13(1), 23–32. 10.3111/13696990903475833 19961361

[r32] Miklowitz, D., Efthimiou, O., Furukawa, T., Scott, J., McLaren, R., Geddes, J., & Cipriani, A. (2021). Adjunctive psychotherapy for bipolar disorder. JAMA Psychiatry, 78(2), 141. 10.1001/jamapsychiatry.2020.2993 33052390 PMC7557716

[r33] Miller, S., Dell’Osso, B., & Ketter, T. (2014). The prevalence and burden of bipolar depression. Journal of Affective Disorders, 169, 3–11. 10.1016/s0165-0327(14)70003-5 25533912

[r34] Morriss, R., Lobban, F., Riste, L., Davies, L., Holland, F., Long, R., … Jones, S. (2016). Clinical effectiveness and acceptability of structured group psychoeducation versus optimised unstructured peer support for patients with remitted bipolar disorder (parades): a pragmatic, multicentre, observer-blind, randomised controlled superiority trial. The Lancet Psychiatry, 3(11), 1029–1038. 10.1016/s2215-0366(16)30302-9 27688021

[r35] National Institute for Health and Care Excellence. (2014). *Bipolar disorder: Assessment and management* [Clinical guideline [CG185]]. NICE. https://www.nice.org.uk/guidance/cg185 31487127

[r36] Oud, M., Mayo-Wilson, E., Braidwood, R., Schulte, P., Jones, S. H., Morriss, R., … Kendall, T. (2016). Psychological interventions for adults with bipolar disorder: Systematic review and meta-analysis. British Journal of Psychiatry, 208(3), 213–222. 10.1192/bjp.bp.114.157123 26932483

[r37] Perich, T., Manicavasagar, V., Mitchell, P., Ball, J., & Hadzi-Pavlovic, D. (2013). A randomized controlled trial of mindfulness-based cognitive therapy for bipolar disorder. Acta Psychiatrica Scandinavica, 127(5), 333–343. 10.1111/acps.12033 23216045

[r38] Riley, R. D., Legha, A., Jackson, D., Morris, T. P., Ensor, J., Snell, K. I. E., … Burke, D. L(2020). One‐Stage Individual Participant Data meta‐analysis models for continuous and binary outcomes: Comparison of treatment coding options and estimation methods. Statistics in Medicine, 39(19), 2536–2555. 10.1002/sim.8555 32394498

[r39] Schünemann, H., Brożek, J., Guyatt, G., & Oxman, A. (Eds.). (2013). GRADE handbook for grading quality of evidence and strength of recommendations. Updated October 2013. The GRADE Working Group. Available from guidelinedevelopment.org/handbook.

[r41] Simon, G., Hunkeler, E., Fireman, B., Lee, J. & Savarino, J. (2007). Risk of suicide attempt and suicide death in patients treated for bipolar disorder. Bipolar Disorders, 9(5), 526–530. 10.1111/j.1399-5618.2007.00408.x 17680924

[r42] Stewart, L. A., Clarke, M., Rovers, M., Riley, R. D., Simmonds, M., Stewart, G., Tierney, J. F. (2015). Preferred reporting items for systematic review and meta-analyses of individual participant data. JAMA, 313(16), 1657–1665. 10.1001/jama.2015.3656 25919529

[r43] van Bronswijk, S., Moopen, N., Beijers, L., Ruhe, H. G., & Peeters, F. (2019). Effectiveness of psychotherapy for treatment-resistant depression: A meta-analysis and meta-regression. Psychological Medicine, 49(3), 366–379. 10.1017/S003329171800199X 30139408

[r44] Van Dijk, S., Jeffrey, J., & Katz, M. (2013). A randomized, controlled, pilot study of dialectical behavior therapy skills in a Psychoeducational group for individuals with bipolar disorder. Journal of Affective Disorders, 145(3), 386–393. 10.1016/j.jad.2012.05.054 22858264

[r45] Yatham, L. N., Kennedy, S. H., Parikh, S. V., Schaffer, A., Bond, D. J., Frey, B. N., …Berk, M. (2018). Canadian network for mood and anxiety treatments (canmat) and International Society for Bipolar Disorders (ısbd) 2018 guidelines for the management of patients with bipolar disorder. Bipolar Disorders, 20(2), 97–170. 10.1111/bdi.12609 29536616 PMC5947163

[r46] Ye, B.-Y., Jiang, Z.-Y., Li, X., Cao, B., Cao, L.-P., Lin, Y., … Miao, G.-D. (2016). Effectiveness of cognitive behavioral therapy in treating bipolar disorder: An updated meta-analysis with randomized controlled trials. Psychiatry and Clinical Neurosciences, 70(8), 351–361. 10.1111/pcn.12399 27177717

[r47] Yilmaz, S., Huguet, A., Kisely, S., Rao, S., Wang, J., Baur, K., …Wright, K. (2022). Do psychological interventions reduce symptoms of depression for patients with bipolar I or II disorder? A meta-analysis. Journal of Affective Disorders, 301, 193–204. 10.1016/j.jad.2021.12.112 35007645

