# Supplementary material for: The clinical efficacy of psychological interventions for bipolar depression: a systematic review and individual patient data (IPD) meta-analysis
Source: Psychol Med. 2025 May 22;55:e154. doi: 10.1017/S0033291725001023 (PMC12115277; doi:10.1017/S0033291725001023)
Supplement: Yilmaz et al. supplementary material 1 — Yilmaz et al. supplementary material [file S0033291725001023sup001.docx]

Appendix 1

**DATA SUPPLY & USE AGREEMENT**

**THIS AGREEMENT** is made on _____________________________ 2020 between:

1. **XXX** whose registered address is at [ ]​ (“**XXX**”); and
2. **UNIVERSITY OF EXETER** whose administrative offices are at Northcote House, The Queen’s Drive, Exeter EX4 4QJ (the ‘**Recipient**’),

each a “**Party**” and collectively the “**Parties**”.

**BACKGROUND**

1. **XXX** has collected and managed the Data (as defined below).
2. The Recipient wishes to obtain the Data for the purposes of one or more research project(s) (the Programme(s) as defined below), to be carried out by Lead Investigator(s) (as defined below) at the Recipient (the “**Purpose**”).
3. **XXX** is willing to provide the Data to the Recipient on the terms and conditions of this Agreement.

**NOW IT IS AGREED** as follows:

1. **DEFINITIONS AND INTERPRETATION**
   1. In this Agreement the following words and expressions shall have the following meanings unless the context expressly requires otherwise:

| “**Co-Investigators**” | means individual investigator(s) identified in Schedule 2 or otherwise agreed in writing by XXX who are based at third-party institutions collaborating with the Recipient on the Programme, and researchers working under their direct supervision. |
| --- | --- |
| “**Co-worker**” | means a researcher from the Recipient working on the Programme under the direct supervision of the Lead Investigator. |
| “**Data**” | means (a) the Original Data; (b) any and all materials, documents, images, designs, plans and information that XXX may provide to the Recipient under or in connection with this Agreement including but not limited to, datasets, databases, photos and videos, as specified further in Schedule 1 as well as any related documentation; (c) any derivative, modified, or improved dataset obtained from or as a result of the use of the whole or any part of any item(s) set out in (a) or (b) above; and (d) any part of any of the foregoing. |
| “**Effective Date**” | means the date of this Agreement. |
| “**Lead Investigator**” | means the individual named as Lead Investigator in Schedule 2. |
| “**Original Data**” | means the dataset(s) to be made available by XXX to the Recipient under this Agreement as described in the attached Schedule 1. |
| “**Programme**” | Means the programme(s) of research, supervised by the Lead Investigator, as described in Schedule 2. |
| “**Term**” | means the term of this Agreement, which shall be a period of seven years from the Effective Date. |

1. **SUPPLY OF DATA TO RECIPIENT**
   1. In consideration of the obligations accepted by the Recipient under this Agreement, XXX shall supply the Data to the Recipient, and shall grant the Recipient an automatic, non-exclusive, royalty-free licence for the use of the Data by the Lead Investigator and any Co-workers for the Purpose; such licence to terminate automatically on expiry or termination of this Agreement.
   2. The licence granted to the Recipient under clause 2.1 shall be non-sublicensable, save that the Recipient shall be permitted to discuss the Data with the Co-Investigators solely for the Purpose; on the basis that such Co-Investigators (or their employer as applicable) have entered into same or similar data supply and use terms, no less onerous than set out in this Agreement. The Recipient shall not have any liability to XXX or any third party, whether by breach or by negligence and whether in contract, tort or otherwise, in relation to the use, transfer, storage or disposal of the Data by such Co-Investigators, their employers or by any third party, to the maximum extent permitted under applicable law.
   3. The Recipient shall ensure that the Data is at all times clearly identified as the property of XXX*.*
   4. The Recipient shall ensure that it provides suitable administrative, technical, and physical safeguards to ensure the confidentiality of the Data and to prevent unauthorised use and access of the Data.
   5. The Recipient shall use the Data solely for the Purpose in accordance with the terms of this Agreement, and not use it for any other purpose or (subject to clause 2.2) supply or expose it to any third party (or permit such supply or exposure), without the prior written consent of XXX.
   6. The Recipient shall comply with all applicable laws and regulations from time to time in its use of the Data.
   7. Wherever applicable, the Recipient shall not use, or permit the use of, the Data in work where it has not first obtained full approval from a properly constituted research ethics committee. If requested by XXX, the Recipient shall supply XXX with a copy of such ethical approval.
   8. Except as required for the Purpose, the Recipient shall not merge or incorporate the Data with any other data set or information, nor change the format of the Data, without the prior written consent of XXX.
   9. Subject to clause 2.2, the Recipient shall not involve any third party in funding or supporting the Programme without the prior written consent of XXX, such consent not to be unreasonably withheld or delayed provided that the terms of such third party support do not conflict with the terms of this Agreement.
   10. Except as expressly permitted under this Agreement in accordance with clause 2.2, the Recipient shall not use the Data or XXX IP for any commercial purpose, including but not limited to commercially-sponsored research or work that is subject to the granting of any rights to a commercial third party, and shall not use or permit the use of any products or processes containing, using or directly deriving from the Data or which are further developments of the Data (including XXX IP) for profit-making or commercial purposes without the prior written consent of XXX.
   11. The Recipient shall make available any raw data generated using the Data if so requested by XXX, which shall regard such raw data as Confidential Information belonging to the Recipient.
2. **INTELLECTUAL PROPERTY AND PUBLICATION**
   1. Nothing in this Agreement shall affect the ownership of intellectual property rights existing prior to this Agreement or generated outside the Programme (“**Background IP**”), including rights in the Data which comprise Background IP belonging to or controlled by XXX. The Data and any copies made thereof, shall at all times remain the property of XXX, and except as expressly stated herein, no licence under any intellectual property belonging to XXX is granted or implied by this Agreement.
   2. New discoveries and inventions created by the Recipient from its use of the Data in the Programme shall belong to the Recipient (“**Recipient IP**”) and constitute part of its Confidential Information, except (i) where such discoveries and inventions purely constitute modifications of XXX’s datasets, designs, images or databases or (ii) where they incorporate, embed or otherwise contain XXX’s Background IP and can only be used by also using all or part of XXX’ Background IP, in which circumstances they shall be owned by XXX (“**Rogers IP**”) and constitute part of its Confidential Information. In the event that the Recipient creates any Recipient IP or XXX IP, it shall promptly bring this to the attention of XXX.
   3. The Recipient hereby grants to XXX an automatic, non-exclusive, fully paid-up, royalty-free, licence to use Recipient IP for its academic, research and educational purposes, and XXX hereby grants the Recipient a non-exclusive, fully paid-up, royalty-free licence to use XXX IP and any Background IP necessary to be able to use such XXX IP for its academic, research and educational purposes.
   4. If any commercial revenues result from the Recipient’s use of the Data (including its use of Recipient IP) XXX shall be entitled to a fair, reasonable and proportionate share of any such revenues.
   5. Nothing in this Agreement shall prevent or unreasonably delay publication of research findings resulting from the use of the Data, which shall be publishable in accordance with normal academic practice according to the process set out in clause 3.8.
   6. Nothing in this Agreement shall prevent or hinder any registered students of the Recipient from submitting for degrees of the Recipient theses based on results obtained during the course of work undertaken Programme; or from following the Recipient’s procedures for examinations and for admission to postgraduate degree status.
   7. When making any publication that refers to the Data, the Recipient shall acknowledge XXX as the source of the Data unless requested to the contrary by XXX, and give it and its researchers due credit for any other contribution they may have made, in accordance with normal academic practice.
   8. The Recipient shall send XXX a draft of all intended publications which describe work carried out using the Data in advance of publication for XXX to review, propose any amendments and to agree with the Recipient on suitable wording to acknowledge its contribution. XXX shall have thirty (30) working days from its receipt of a draft of a proposed publication to comment on the publication and/or request a delay or amendment to ensure that information regarding the Data is handled in accordance with applicable laws and regulations, to obtain protection for its intellectual property or to require that any of its Confidential Information is removed, genericised or otherwise amended. Any delay to protect intellectual property shall be only for the minimum time necessary to obtain such protection. If XXX makes a reasonable request to amend or delay a publication, the Recipient, Lead Investigator and any Co-workers shall amend or delay that publication as requested by Rogers or else enter into discussions with XXX to agree between themselves in good faith a mutually acceptable form of wording for the publication.
   9. If no notification under clause 3.8 is received from XXX by the Recipient within thirty (30) working days from XXX’ receipt of the proposed publication, the Recipient shall be free to assume that XXX has no objection to the proposed publication.
3. **CONFIDENTIALITY**
   1. In the event of one Party making available to the other Party (the “**Receiving Party**”) confidential or proprietary information relating to its scientific, business or other activities, including Background IP and information supplied in any form supporting, describing or otherwise relating to the Data, such as data, designs, datasets, plans, drawings and invention disclosures related to the Data (“**Confidential Information**”), the Receiving Party shall:
4. hold it in strict confidence,
5. only use it for the purpose for which it is disclosed,
6. only disclose it to its staff and students on a strict ‘need to know’ basis, after establishing that they are aware of their obligations under this clause, and
7. not disclose it to any third party.
   1. The obligations of confidence referred to in this Clause 3 shall not extend to any information which a Party can clearly demonstrate by written record:
8. was known to it prior to disclosure; or
9. was or becomes part of the public domain through no fault of the Receiving Party; or
10. becomes lawfully available to the Receiving Party from an unconnected third party who is under no obligation of confidentiality in respect thereof, or with the lawful right to make such disclosure; or
11. has been independently developed or conceived for or by it without reference to the other Party’s Confidential Information; or
12. it is required to disclose by law or a regulatory body, in which circumstances the Receiving Party shall wherever practicable give reasonable advance notice of the intended disclosure to the other Party, and the relaxation of the obligations of confidentiality shall apply only for as long as is necessary to comply with the relevant law or regulatory requirement and solely for the purposes of such compliance.
    1. The above obligations of confidentiality shall survive expiry or termination of this Agreement by five (5) years.
    2. Except as provided for herein, neither Party shall use the other’s name, crest, logo or trademarks, or the name of any of its staff or students without the express written permission of that Party or individual, except that nothing in this clause shall restrict, delay, impede or prevent a Party from using the other Party’s name when making disclosures under freedom of information legislation or in its own reports or internal literature.
13. **WARRANTIES & LIABILITIES**
    1. All Data delivered under this Agreement is supplied ‘as is’ and XXX makes no representations, undertakings or warranties of any kind, either express or implied regarding the Data, and by way of example without limiting the foregoing, there are no express or implied warranties or undertakings of merchantability, that (i) the Data is of satisfactory quality or fit for a particular purpose, or (ii) the use of the Data will not infringe any patent, copyright, trademark, or other proprietary right owned by a third party. The terms and conditions herein are agreed to be *in lieu* of any warranties, obligations or conditions implied by law, trade usage, custom or otherwise as to the merchantable quality or the fitness for any particular purpose of the Data.
    2. XXX shall have no liability to the Recipient or any third party, whether by breach or by negligence and whether in contract, tort or otherwise, in relation to the use, transfer, storage or disposal of the Data by the Recipient or by any third party, to the maximum extent permitted under applicable law.
    3. The Parties undertake to make no claim in connection with this Agreement or its subject matter against any employees, students, agents or appointees of the other Party (apart from claims based on fraud or wilful misconduct). This undertaking is intended to give protection to individual researchers: it does not prejudice any right which a Party might have to claim against any other Party.
    4. Notwithstanding any other provision of this Agreement:
14. no Party shall have any liability to the other for claims and losses for loss of income, profits, turnover, business, opportunity, reputation, goodwill, economic loss, indirect loss or consequential loss, no matter how arising and whether by breach or by negligence and whether in contract, tort or otherwise; and
15. in any event, the maximum liability of any Party under or otherwise in connection with this Agreement or its subject matter shall not exceed the sum of £10,000.
16. **TERMINATION**
    1. This Agreement may be terminated before the end of the Term by either Party subject to written notice of a minimum of thirty (30) days.
    2. Either Party may terminate this Agreement by giving notice in writing to that effect to the other Party if:
17. the other Party is in breach of its obligations under this Agreement and fails to remedy such breach within thirty (30) working days of receiving notice from the other Party that clearly identifies and gives details of the breach; or
18. the other Party enters into bankruptcy or liquidation, has a receiver, liquidator or administrator appointed over the whole or any part of its assets, becomes insolvent or is otherwise unable to pay its debts as they fall due (with the exception of liquidation for the specific purpose of an amalgamation, reconstruction or other reorganisation such that the body resulting from the reorganisation agrees to be bound by and to assume the obligations imposed on the relevant Party herein *mutatis mutandis*);
    1. At the end of the Term or upon termination of this Agreement the licences and rights granted to the Recipient herein to use Data or Confidential Information shall automatically terminate unless specifically renewed, and the Recipient, Lead Investigator and any Co-workers shall cease to use the Data and any Confidential Information, and, if instructed by XXX, shall to the extent practically possible delete or destroy all Confidential Information, Data and any copies thereof in their possession or under their control.
    2. The Term may be extended subject to the agreement of XXX, and any such extension shall be effected by means of a formal amendment to this Agreement.
    3. Termination or expiry of this Agreement shall not affect the survival of any clauses or provisions herein which are stated, or which by their nature are intended, to continue after termination or expiry.
19. **GENERAL**
    1. No Party shall assign, transfer or subcontract this Agreement or any of its obligations, duties and benefits hereunder without the prior consent of the other Party, which shall not unreasonably be withheld.
    2. The Recipient shall ensure that any employee or representative of the Recipient who has access to the Data is made aware of the terms of this Agreement and that the Lead Investigator and any Co-workers handle, use, store and dispose of the Data in accordance with the provisions of this Agreement.
    3. Any notices served under this Agreement shall be made in writing and sent to each Party at the address shown below, or to such other address as a Party may from time to time have notified each of the other Parties as being its address for such correspondence:

**XXX’ address for notices:**

**For legal notices:** [ ]

**For technical notices:** [ ]

**Recipient’s address for notices:**

**For legal notices**: Chrysten Cole, General Counsel and Deputy Director of Compliance, Governance and Risk, Lafrowda House, St German’s Road, Exeter, Devon, EX4 6TL

**For technical notices**: Kim Wright, Washington Singer Laboratories 117, University of Exeter, Perry Road, Prince of Wales Road, Exeter, EX4 4QG, UK

- 1. The terms and conditions set out herein are intended to supplement any legal or regulatory requirements governing the handling, use, transfer, storage and disposal of Data that may be in force from time to time and not intended to conflict or contradict them but should any conflict or contradiction arise between the terms of this Agreement and any relevant legal or regulatory requirements, the legal or regulatory requirements shall take precedence over any term of this Agreement. If any part or any provision of this Agreement proves to any extent to be invalid, illegal or unenforceable in law, the remainder of such provision and all other provisions of this Agreement shall remain valid and enforceable to the fullest extent permissible by law and regulation, and such provision shall be deemed to be omitted from this Agreement to the extent of such invalidity, illegality or unenforceability. The remainder of this Agreement shall continue in full force and effect and the Parties shall negotiate in good faith to replace the invalid, illegal or unenforceable provision with a valid, legal and enforceable provision which has an effect as close as possible to the provision or terms being replaced.
  2. In the event of any difference, dispute or question, arising from this Agreement or the Programme, the Parties will first attempt to resolve the matter informally through designated senior representatives of each Party to the dispute, who are not otherwise involved with the Programme. Should the Parties still be unable to reach agreement between themselves within thirty (30) working days, they shall refer the matter promptly for resolution using Alternative Dispute Resolution techniques by either (i) the Centre for Effective Dispute Resolution if both Parties are based in the UK or (ii) the International Chamber of Commerce if one of the Parties is based outside the UK. Any decision reached in this way shall be final and binding upon the Parties involved. Nothing in this clause shall prevent or impede XXX from being free to apply for interim relief in any court of competent jurisdiction seeking to require the Recipient to cease using and/or return Data or Confidential Information in the event of a breach of this Agreement.
  3. This Agreement, including any schedules, supersedes and terminates all other agreements, terms, understandings and representations regarding the Data whether written, oral, express or implied between the Parties and constitutes the entire agreement between the Parties concerning the Data and the sole basis on which they have entered into this Agreement.
  4. The clause headings in this Agreement are used for convenience only and shall not affect its interpretation or construction.
  5. This Agreement shall be binding upon and shall inure to the benefit of the Parties and their respective successors in title and assigns, and any amendments to this Agreement must be agreed in writing by the Parties, their successors in title or their assigns.
  6. The validity, construction and performance of this Agreement shall be governed by the laws of England and Wales and it shall be subject to the jurisdiction of the English courts.

**EXECUTED** as an agreement:

**SIGNED** for and on behalf of **XXX**

Name:

Position:

Signature:

Date:

**SIGNED** for and on behalf of **the Recipient**

Name:

Position:

Signature:

Date:

**Acknowledged** by the **Lead Investigator**

Name:

Signature:

Date:

**SCHEDULE 1: DESCRIPTION OF DATA**

**SCHEDULE 2: DESCRIPTION OF PURPOSE**

**Title of Programme:**

**Name of Lead Investigator:**

**Name and Institution of Co-Investigators:**

**Description of Programme:**
