# Supplementary material for: The clinical efficacy of psychological interventions for bipolar depression: a systematic review and individual patient data (IPD) meta-analysis
Source: Psychol Med. 2025 May 22;55:e154. doi: 10.1017/S0033291725001023 (PMC12115277; doi:10.1017/S0033291725001023)
Supplement: Yilmaz et al. supplementary material 2 — Yilmaz et al. supplementary material [file S0033291725001023sup002.docx]

**Table S1** *A summary of selected key characteristics of included versus excluded studies*

| Primary intervention target | Number of studies excluded versus included | Depression status at baseline | Number of studies excluded versus included | Treatment group | Number of studies excluded versus included | Comparator group | Number of studies excluded versus included |
| --- | --- | --- | --- | --- | --- | --- | --- |
| Relapse prevention | 8/2 | Not depressed | 7/4 | Psychoeducation | 14/1 | Usual care | 14/3 |
| Acute depression | 6/- | Not specified | 16/2 | CBT | 9/2 | Placebo | 6/1 |
| Unclear/Unspecified | 2/3 | Depressed | 2/- | MBCT | 2/1 | Active control | 2/1 |
| Quality of life | 3/- |  |  | DBT | -/1 | Waiting list | 3/1 |
| Other | 6/1 |  |  | IPSRT | -/1 |  |  |

**Table S2**

*A summary of multilevel model output showing the relationship between condition allocation and treatment outcome*

| **Parameter** | **Estimate(SE)** | **Sig.** | **95% CI** |
| --- | --- | --- | --- |
| Intercept | 7.41(.53) | .000 | [6.36,8.47] |
| Condition allocation | 1.86(.65) | .005 | [.58,3.15] |

**Table S3**

*A summary of multilevel model outputs showing the relationship between condition allocation, baseline depression and treatment outcome*

| **Parameter** | **Estimate(SE)** | **Sig.** | **95% CI** |
| --- | --- | --- | --- |
| Intercept | 3.20(.66) | <.001 | [1.91,4.48] |
| Condition allocation | 1.80(.67) | .007 | [.49,3.10] |
| Baseline depression | .43(.04) | .000 | [.34,.51] |

**Table S4**

*A summary of multilevel model outputs showing the relationship between condition allocation, baseline depression, interaction between them, and treatment outcome*

| **Parameter** | **Estimate(SE)** | **Sig.** | **95% CI** |
| --- | --- | --- | --- |
| Intercept | 1.80(1.60) | .261 | [-1.34,.94] |
| Condition allocation | 1.58(2.11) | .454 | [-2.56,5.72] |
| Baseline depression | .58(.13) | <.001 | [.32,.84] |
| Condition allocation*  Baseline depression | .05(.17) | .762 | [-.28,.38] |

**Table S5**

*A summary of multilevel model output showing the relationship between condition allocation and treatment outcome*

| **Parameter** | **Estimate(SE)** | **Sig.** | **95% CI** |
| --- | --- | --- | --- |
| Intercept | 7.81(.95) | <.001 | [5.95,9.67] |
| Condition allocation | 2.72(1.36) | .046 | [.05,5.39] |

**Table S6**

*A summary of multilevel model outputs showing the relationship between condition allocation, baseline depression and treatment outcome*

| **Parameter** | **Estimate(SE)** | **Sig.** | **95% CI** |
| --- | --- | --- | --- |
| Intercept | 1.50(1.21) | .216 | [-.88,3.88] |
| Condition allocation | 2.13(1.15) | .065 | [-.13,4.39] |
| Baseline depression | .61(.08) | <.001 | [.45,.78] |

**Table S7**

*A summary of multilevel model outputs showing the relationship between condition allocation, baseline depression, interaction between them, and treatment outcome*

| **Parameter** | **Estimate(SE)** | **Sig.** | **95% CI** |
| --- | --- | --- | --- |
| Intercept | 3.35(.76) | <.001 | [1.85,4.85] |
| Condition allocation | 1.50(1.03) | .144 | [-.51,3.52] |
| Baseline depression | .42(.07) | <.001 | [.27,.56] |
| Condition allocation*  Baseline depression | .03(.09) | .752 | [-.14,.20] |
